# Differential modulation of movement speed with state-dependent deep brain stimulation in Parkinson’s disease

**DOI:** 10.1126/sciadv.adx6849

**Published:** 2025-09-10

**Authors:** Alessia Cavallo, Richard M. Köhler, Johannes L. Busch, Jeroen G. V. Habets, Timon Merk, Patricia Zvarova, Jojo Vanhoecke, Thomas S. Binns, Bassam Al-Fatly, Ana Luisa de Almeida Marcelino, Natasha Darcy, Gerd-Helge Schneider, Patricia Krause, Andreas Horn, Katharina Faust, Damian M. Herz, Eric Yttri, Hayriye Cagnan, Andrea A. Kühn, Wolf-Julian Neumann

**Affiliations:** ^1^ Movement Disorder and Neuromodulation Unit, Department of Neurology, Charité–Universitätsmedizin Berlin, corporate member of Freie Universität Berlin and Humboldt-Universität zu Berlin, Berlin, Germany.; ^2^ Bernstein Center for Computational Neuroscience Berlin, Berlin, Germany.; ^3^ Einstein Center for Neurosciences Berlin, Charité–Universitätsmedizin Berlin, corporate member of Freie Universität Berlin and Humboldt-Universität zu Berlin, Berlin, Germany.; ^4^ Department of Neurosurgery, Baylor College of Medicine, Houston, TX, USA.; ^5^ Department of Electrical & Computer Engineering, Rice University, Houston, TX, USA.; ^6^ Institute for Network Stimulation, Department of Stereotactic and Functional Neurosurgery, University Hospital Cologne, Cologne, Germany.; ^7^ Department of Neurosurgery, Charité–Universitätsmedizin Berlin, corporate member of Freie Universität Berlin and Humboldt-Universität zu Berlin, Berlin, Germany.; ^8^ Center for Brain Circuit Therapeutics, Department of Neurology, Brigham & Women’s Hospital, Harvard Medical School, Boston, MA, USA.; ^9^ MGH Neurosurgery & Center for Neurotechnology and Neurorecovery (CNTR) at MGH Neurology Massachusetts General Hospital, Harvard Medical School, Boston, MA, USA.; ^10^ Department of Neurosurgery, University Hospital Düsseldorf, Heinrich Heine University Düsseldorf, Düsseldorf, Germany.; ^11^ Department of Neurology, University Hospital Heidelberg, Heidelberg, Germany.; ^12^ Department of Biological Sciences, Neuroscience Institute, Carnegie Mellon University, Pittsburgh, PA, USA.; ^13^ Department of Bioengineering, Imperial College London, London, UK.; ^14^ NeuroCure Clinical Research Centre, Charité–Universitätsmedizin Berlin, corporate member of Freie Universität Berlin and Humboldt-Universität zu Berlin, Berlin, Germany.; ^15^ Berlin School of Mind and Brain, Humboldt-Universität zu Berlin, Berlin, Germany.

## Abstract

Subthalamic deep brain stimulation (STN-DBS) provides unprecedented spatiotemporal precision for the treatment of Parkinson’s disease (PD), allowing for direct real-time state-specific adjustments. Inspired by findings from optogenetic stimulation in mice, we hypothesized that STN-DBS can mimic dopaminergic reinforcement of ongoing movement kinematics during stimulation. To investigate this hypothesis, we delivered DBS bursts during particularly fast and slow movements in 24 patients with PD. Our findings reveal that DBS during fast movements enhanced future movement speed more than DBS during slow movements, raising movement speed to the level of healthy controls. To understand which brain circuits mediate this neurophysiological mechanism, we investigated the behavioral effects using magnetic resonance imaging connectomics and motor cortex electrocorticography. Last, we demonstrate that machine learning–based brain signal decoding can be used to predict continuous movement speed for fully embedded state-dependent closed-loop algorithms. Our findings provide important evidence for reinforcement-based DBS circuit mechanisms that may inspire previously unexplored treatment avenues for dopaminergic disorders.

## Teaser

STN-DBS during fast movements boosts future motor vigor, mimicking dopamine-like reinforcement in Parkinson’s disease.

## INTRODUCTION

Subthalamic deep brain stimulation (STN-DBS) is an effective treatment for Parkinson’s disease (PD), one of the fastest-growing neurodegenerative disorders (*1*, *2*). Although stimulation parameters are traditionally kept unchanged over months or years, STN-DBS holds the potential to be adapted within milliseconds, allowing neuromodulation with unprecedented temporal precision (*3*). This precision may be used to selectively modulate certain brain states, potentially increasing treatment efficacy beyond current chronic STN-DBS approaches. Over the past decade, subthalamic beta-band activity has been identified as a biomarker of PD symptom severity (*4*–*6*), leading to the development of beta-based adaptive DBS, which ties stimulation strength to concurrent symptom severity (*7*–*9*). In the context of adaptive DBS, however, it is unknown how concurrent behavior, such as voluntary movement, during adaptive stimulation episodes, interacts with the stimulation and potentially modulates its effect. Recent studies in rodents have revealed that transient basal ganglia pathway modulation influences future behavior in a state-specific manner (*10*, *11*). Using closed-loop speed-triggered optogenetic stimulation of direct pathway spiny projection neurons in the dorsal striatum, Yttri and Dudman demonstrated that the speed during stimulation was decisive for the behavioral aftereffect (*10*). Namely, stimulation during the fastest movements led to a subsequent increase in movement speed, whereas stimulation during the slowest movements led to a decrease in speed. A similar reinforcement of movement speed was reported by Markowitz *et al.*, who additionally showed that both the speed of movements and their occurrence could be enhanced by movement-selective optogenetic stimulation of dopamine-releasing neurons targeting the dorsal striatum (*11*). Although these studies target different neuronal populations, both increase the strength of the direct basal ganglia pathway, shifting the balance away from the indirect pathway. STN-DBS applied in PD is now thought to modulate indirect pathway activity and net inhibitory output through suppression of hyperdirect input and local synaptic depression (*12*, *13*). On a circuit level, transient STN-DBS may thus have similar effects to transient optogenetic activation of striatal direct pathway or dopaminergic neurons (*14*) and could lead to a phasic reduction of pallidothalamic inhibition. With this in mind, we attempt to reproduce the effects of state-specific optogenetic stimulation in human patients using STN-DBS. To do so, we translated the experimental approach presented by Yttri and Dudman into a motor state–dependent speed-selective STN-DBS paradigm for PD. Slowness of movement, also termed bradykinesia, is a cardinal symptom of PD (*15*, *16*). We hypothesized that state-dependent stimulation may have stronger antibradykinetic effects when applied during fast movements compared to slow movements. Stimulation of fast but not slow movements successfully counteracted bradykinesia, providing initial evidence that STN-DBS motor effects are acutely motor state–dependent. Using magnetic resonance imaging (MRI)–based connectomics, we identified critical brain networks associated with the speed-specific stimulation effects, revealing the supplementary motor cortex and putamen as important hubs. Last, we uncovered stimulation-induced oscillatory changes in electrocorticography (ECoG) signals and demonstrated their utility in movement decoding for potential future applications of movement-responsive closed-loop STN-DBS.

## RESULTS

### Speed-selective STN-DBS

**Fig. 1.**
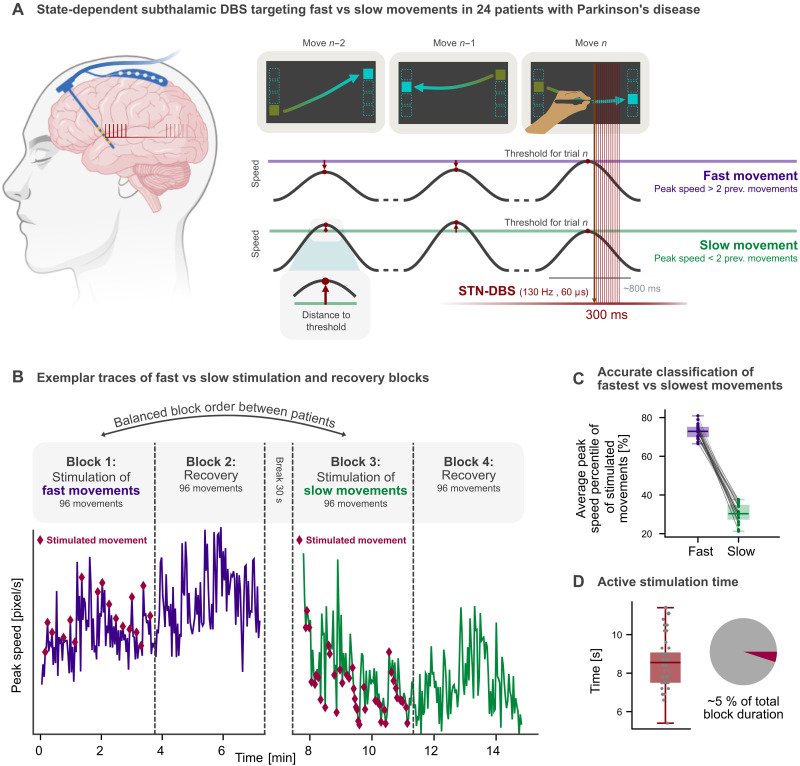
Overview of behavioral task and stimulation algorithm. Participants engaged in a visuomotor tablet task (**A**) during which they had to move a pen to a colored rectangle appearing on either side of the tablet. In 24 patients with PD OFF dopaminergic medication, 300-ms bursts of subthalamic DBS were applied bilaterally either during movements classified as fast or slow. Stimulation was triggered in accordance with the peak speed, which was used for the classification of fast (purple) and slow (green) conditions. In the case of a peak speed higher than that of the previous two movements, the ongoing movement was classified as fast, whereas a peak speed lower than that of the previous two movements led to a classification as slow. Movements lasted 812 ± 115 ms, and stimulation bursts started 66 ms after the peak speed on average. (**B**) Patients underwent four blocks of 96 movements each. During blocks 1 and 3, either slow or fast movements were stimulated (red diamonds mark stimulated movements for one exemplary patient), followed by recovery blocks 2 and 4, during which no stimulation was administered. The order of stimulation conditions was balanced across patients. (**C**) The average peak speed of the stimulated slow and fast movements lies in the 21 to 37% and 66 to 80% of all movements in the stimulation block, respectively. (**D**) The average total stimulation time of 8.51 ± 1.46 s resulted in stimulation being present during ~5% of the stimulation block.

To investigate the effects of speed-selective adaptive stimulation, we recruited 24 patients with PD undergoing STN-DBS. Experiments were performed in the perioperative period before implantation of the pulse generator, which allowed closed-loop control of STN-DBS amplitude through externalized leads. Patients were tested in the clinical OFF state, after 12 hours of withdrawal of all dopaminergic medication. Before the experimental paradigm, we determined clinically effective stimulation contacts and amplitudes through a monopolar review. In addition, we recruited 14 healthy age-matched control subjects to provide a behavioral reference. All subjects performed a visuomotor tablet task on a hybrid monitor and digitizing tablet. In this task, a colored rectangle alternately appeared on the left and right side of a tablet screen, and subjects performed a forearm movement to guide a touchscreen pen to the target rectangle (Fig. 1A). After a short familiarization period, speed-selective DBS targeting fast or slow movements was applied during a block of 96 movements, followed by a recovery block of the same length during which no stimulation was applied (Fig. 1B). The order of conditions was balanced across patients, who were blinded to the stimulation condition. Healthy participants performed the task without stimulation. The paradigm approximately translated the experimental approach of speed-selective optogenetic stimulation in rodents (*10*) to investigate the behavioral aftereffects of speed-selective STN-DBS in patients with PD. For that, we used real-time movement speed tracking to deliver speed-selective stimulation in patients with PD, eliciting 300-ms bursts of bilateral 130-Hz STN-DBS at clinically effective amplitudes (table S1) during relatively fast or slow movements. The stimulation control algorithm was optimized to stay unbiased by naturally occurring fluctuations in the patient’s motor behavior, such as the bradykinetic decrement, and, at the same time, approximated the same portion of stimulated movements in the optogenetic experiment that inspired this study (one-third). In brief, fast and slow movements were classified on the basis of their peak speed relative to the previous two movements. If the peak speed of the ongoing movement exceeded the speed of the previous two movements, it was classified as fast; if it fell under the previous two peak speeds, it was classified as slow (Fig. 1A). This dynamic classification algorithm had an accuracy of 96% across conditions and patients (table S2) and resulted in the stimulation of 26.3 ± 3.2% of movements in the fast stimulation block and 32.8 ± 4.5% of movements during the slow stimulation block across patients. Post hoc analysis revealed that, across patients, the average peak speed of the stimulated slow and fast movements lies in the 21 to 37% and 66 to 80% percentiles of all movements in the stimulation block, respectively (Fig. 1C). Thus, the dynamic classification of fast and slow movements successfully targeted the upper and lower third of the distribution per stimulation block, respectively. Given the short duration of the stimulation bursts, this translated into an active stimulation time of only 8.51 ± 1.46 s across the stimulation blocks, approximately 5% of the total block duration (Fig. 1D).

### Movement state–dependent STN-DBS differentially modulates movement speed

**Fig. 2.**
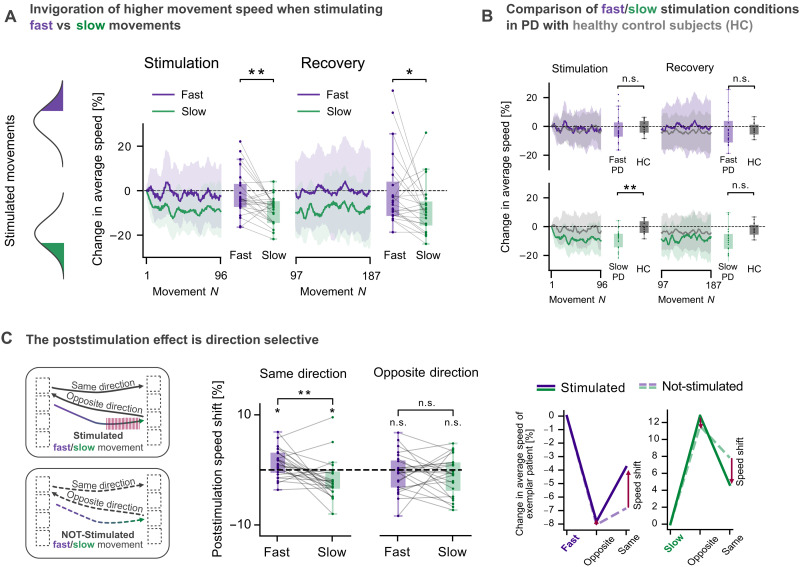
The effect of subthalamic DBS on future movement speed depends on the speed of the stimulated movement. (**A**) 300-ms STN-DBS had stronger antibradykinetic effects with less decline in the overall movement speed if applied during fast compared to slow movements (*n* = 24, *P* < 0.01). This less pronounced speed decline could also be observed in the recovery block, following the same pattern, although no stimulation was applied (*P* < 0.05). (**B**) Comparison with age-matched healthy control subjects showed no significant difference in speed changes between patients with PD in the fast stimulation block and the control group (*n*_control = 14, *P* > 0.05). However, when slow movements were stimulated, patients with PD experienced a significantly greater decline in speed compared to the controls (*P* < 0.05). (**C**) Inspection of poststimulation movements revealed a stimulation-induced shift toward the speed of the stimulated movement only when subsequent movement direction was concordant with the direction during stimulation (same direction *P* < 0.01; opposite direction *P* > 0.05). Compared to not-stimulated fast/slow movements extracted from the recovery block, stimulation of fast movements led to an increase, whereas stimulation of slow movements resulted in a decrease in subsequent speed (both *P* < 0.05). Two-sided permutation tests (100,000 permutations) were used: paired-sample for within-subject, independent sample for between-subject comparisons, and sign-flipping for testing against zero. n.s., not significant; *P < 0.05; **P < 0.01.

First, we aimed to address the hypothesis that stimulation effects on motor output depend on the ongoing motor behavior, rendering STN-DBS acutely motor state–dependent. Bradykinesia, or slowness of movement, is a disease-defining feature of PD, leading to the deterioration of amplitude and velocity of movements with repetition. If our hypothesis held true, stimulating only the fastest movements could reinforce high movement speeds and thus counteract bradykinesia, whereas stimulating particularly slow movements may have no or even an aggravating effect on movement slowness. To quantify the modulation of speed, we extracted the average speed for each movement and calculated the change in percent with respect to the five initial movements of each stimulation block. For healthy control subjects, because no stimulation was applied, we averaged the change in speed over blocks. We found that short bursts of STN-DBS during fast compared to slow movements had a stronger antibradykinetic effect on the overall movement speed, i.e., speed declined less strongly in the block in which fast movements were targeted compared to the block in which slow movements were stimulated (Fig. 2A; *P* < 0.01). Comparison with healthy age-matched control subjects revealed that the changes in speed between patients with PD in the fast stimulation block and healthy subjects were indistinguishable (Fig. 2B; *P* > 0.05). If slow movements were stimulated, however, the speed of patients with PD declined more strongly than that of healthy controls (Fig. 2B; *P* < 0.01). This demonstrates that STN-DBS effects on motor control depend on the exhibited movement kinematics at the time stimulation is applied. To test whether the observed effects outlasted the stimulation block, we conducted the same comparisons for the recovery block, during which no stimulation was applied. The difference between stimulation conditions remained significant (Fig. 2A; *P* < 0.05), with a more positive change in speed in the recovery block following the stimulation of fast movements compared to the stimulation of slow movements. No difference was found between the change in speed of healthy control subjects and that of patients with PD in the recovery block following either condition (Fig. 2B; *P* > 0.05).

Our results demonstrate that motor state–dependent STN-DBS differentially modulates movement speed. Stimulation during fast but not slow movements had a significant antibradykinetic effect despite stimulation being active for only ~5% of the overall movement time. Notably, stimulation-induced effects outlasted the stimulation block, suggesting plasticity-like mechanisms, which could be exploited in future applications of closed-loop DBS for the treatment of PD and other brain disorders.

### Differential speed modulation effects are reach-direction selective

After analyzing block-averaged changes in movement speed, we aimed to characterize the effect of stimulation on individually identified subsequent movements, both in the same and the opposite direction. To do so, we calculated the change in speed for the two following movements, normalized to the speed of the stimulated movement. To reveal stimulation-induced shifts in movement speed, we extracted fast and slow movements from the recovery block, during which no stimulation was applied, according to the same algorithm used for speed-selective stimulation. We then calculated the poststimulation speed shift as the difference between the change in speed following stimulated and not-stimulated movements. We found that stimulation during fast movements resulted in increased movement speed compared to stimulation of slow movements (Fig. 2C; *P* < 0.01). One-sample tests confirmed the expected differential effects, showing significantly increased speed following stimulation of fast movements (*P* < 0.05) and significantly decreased speed following stimulation of slow movements (*P* < 0.05). This differential modulation was limited to the subsequent movement in the same direction and was not apparent for the movement in the opposite direction (*P* > 0.05).

These results indicate that the observed effects are selective for the reach direction at the time point of stimulation, suggesting that state-specific reinforcing effects on motor performance may be movement trajectory specific.

### MRI connectivity accounts for speed-dependent stimulation effects

**Fig. 3.**
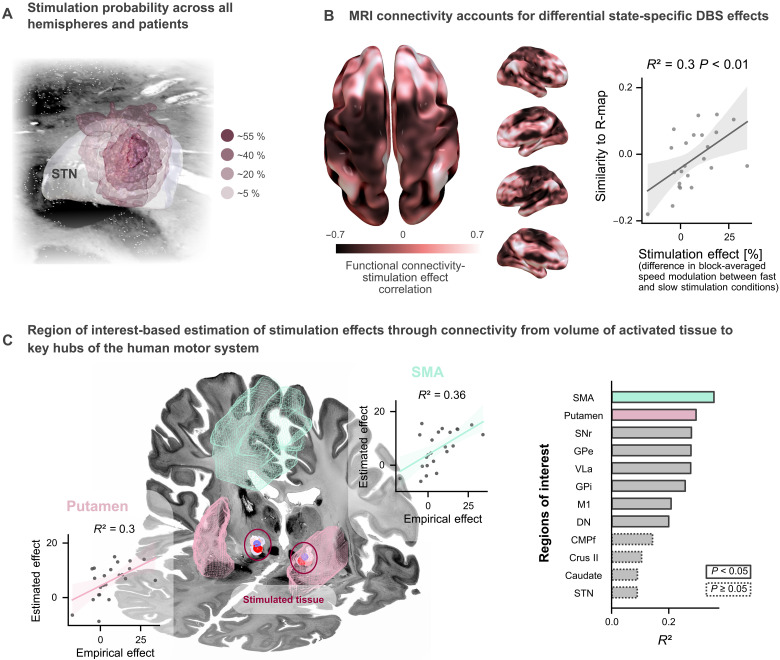
DBS connectivity accounts for speed-dependent stimulation effects. (**A**) Stimulation probability averaged across hemispheres and patients derived from an overlap of stimulation volumes indicates the highest stimulation probability in the dorsolateral motor part of the subthalamic nucleus. (**B**) The whole-brain voxel-wise correlation map demonstrates the optimal functional connectivity profile for speed-selective DBS effects on movement speed. The speed-selective DBS effect was quantified as the difference in block-averaged speed modulation between fast and slow movements, thus the difference between blocks 1 and 2 (stimulation of fast movements and subsequent recovery) and blocks 3 and 4 (stimulation of slow movements and subsequent recovery). The more the individual whole-brain functional connectivity profile seeding at the bilateral stimulation volumes matched the “optimal” map, the higher the difference between stimulation conditions (*n* = 23, *R*^2^ = 0.3, *P* < 0.01, leave-one-subject-out cross-validated). (**C**) In an additional region of interest–based analysis, functional connectivity to the SMA (linear regressor; *R*^2^ = 0.36, *P* < 0.05) could best predict the observed speed-dependent effect, followed by connectivity to the putamen (linear regressor; *R*^2^ = 0.3, *P* < 0.05) and other basal ganglia nuclei, thalamus, M1, and cerebellar dentate nucleus, which provided less predictive but still significant estimations (separate linear regression models; solid line indicates a significant regressor, and dotted lines indicate nonsignificance, *P* values FDR corrected). Pearson’s correlation was used. SNr, substantia nigra pars reticulata; GPe, globus pallidus externus; VLa, basal ganglia receiving the ventrolateral anterior thalamic nucleus; GPi, globus pallidus internus; M1, primary motor cortex; DN, cerebellar dentate nucleus; CMPf, intralaminar centromedian/parafascicular thalamic nucleus; STN, subthalamic nucleus; Crus II, cerebellar Crus II.

After examining the behavioral effects of stimulation, we aimed to identify critical brain networks associated with the state-dependent modulation of STN-DBS on movement speed. We used functional magnetic resonance imaging (fMRI)–based connectomics to elucidate whether the extent of the effect was mediated by the circuit architecture, connecting the stimulated tissue in the subthalamic nucleus to the rest of the brain. To do so, we followed the analysis pipeline provided by Lead-DBS, which has been used in the past to explain variance in clinical and behavioral outcomes following DBS (*17*–*21*). First, we localized DBS electrodes and determined the volume of activated tissue in each hemisphere for 23 patients on the basis of an established methodology (Fig. 3A; one subject had an incompatible electrode type) (*22*). We then used an openly available connectome derived from resting-state fMRI images from patients with PD to calculate patient-individual connectivity maps, seeding from the stimulation volumes. For each patient, we determined the extent of the state-dependent stimulation effect as the difference in block-averaged speed modulation between fast versus slow stimulation conditions. For each voxel in the brain, we then correlated the patient-individual effect strengths with the individual functional connectivity, yielding a whole-brain correlation map (Fig. 3B). This map constitutes the “optimal” functional connectivity profile associated with the strongest stimulation effect. The similarity of patient-specific connectivity maps with this “optimal” map could significantly account for speed-dependent stimulation effects in left-out patients (Fig. 3B, *R*^2^ = 0.3, *P* < 0.01, leave-one-patient-out cross-validation). Thus, the stronger an electrode was connected to the identified network, the stronger the state-dependent stimulation was in our experiment. To gather further insights on the key hubs underlying this correlation, we conducted a region of interest analysis focused on the human motor network, for which we selected 12 brain regions comprising the cortical and subcortical motor system, including motor cortex, thalamic, basal ganglia, and cerebellar connections (*23*–*26*). For each region, a linear regression model predicting the stimulation effect was trained using the average functional connectivity seeding at the stimulation volumes. Functional connectivity to the supplementary motor area (SMA) best accounted for speed-dependent differences in DBS effects, explaining 36% of the observed variance [Fig. 3C; *R*^2^ = 0.36, *P* < 0.05, false discovery rate (FDR) corrected], followed by basal ganglia (putamen, substantia nigra, and globus pallidus externus and internus), the ventrolateral anterior nucleus (VLa) of thalamus, and the dentate nucleus of cerebellum (all *P* < 0.05, FDR corrected).

These results suggest that our speed-selective neuromodulation effect critically depends on the stimulation electrode being connected to a specific brain network, which includes the SMA, basal ganglia, and basal ganglia receiving the VLa.

### State-specific neurostimulation modulates motor cortical beta oscillations

**Fig. 4.**
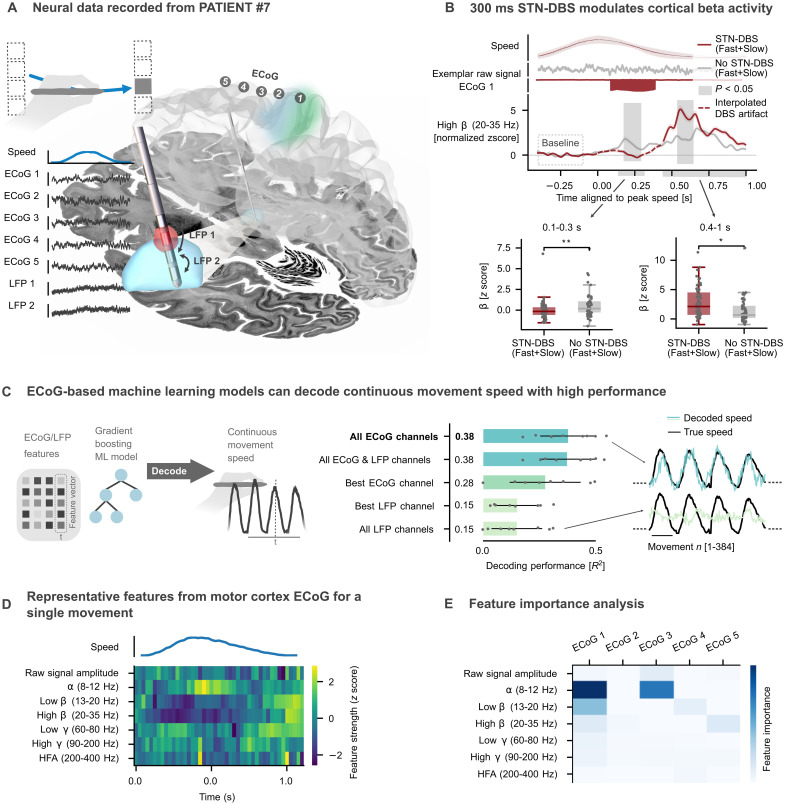
Motor cortex ECoG from patient #7. (**A**) ECoG and DBS/LFP lead placement in the right hemisphere of patient #7, who performed the task with their left hand. The red marker indicates the stimulation contact used for 300-ms bursts of speed-selective stimulation at 2 mA, 130-Hz frequency, and 60-μs pulse width. Common-average ECoG and bipolar LFP referencing resulted in seven intracranial electroencephalography (EEG) channels. (**B**) Comparison of motor cortex beta activity (ECoG channel 1) during movements with stimulation (fast+slow pooled) compared to speed-matched trials without stimulation demonstrates a stimulation-induced decrease in perimovement beta power (*n* = 59, *P* < 0.01) and a stimulation-induced increase in postmovement beta synchronization (*P* < 0.05). Pooling of fast and slow movement trials avoided a potential bias from differences in kinematics and movement timing. Shaded colored areas show the SEM. Shaded light gray areas indicate clusters of significant differences. Significant differences across averages of movement (0.1 to 0.3 s) and postmovement (0.4 to 1.0 s) periods are shown as box plots with individual beta amplitudes below. (**C**) Continuously recorded movement speed could be successfully decoded using LFP and ECoG signals in a gradient boosting machine learning (ML) model (CatBoost). The highest performance was achieved by combining all ECoG channels (*R*^2^ = 0.38 ± 0.13, hyperparameters optimized and model validated through fourfold inner and eightfold outer nested cross-validation). (**D**) Exemplar trace of oscillatory features and the raw signal amplitude extracted from an exemplar channel and movement using py**_**neuromodulation. (**E**) Analysis of ML model feature importance (prediction value change) reveals alpha and low beta power as the most informative features for movement speed decoding (optimal decoder for an exemplar outer fold). n.s., not significant. Two-sided independent-sample permutation tests (100,000 permutations) were used. *P < 0.05; **P < 0.01.

To study the neurophysiological underpinnings of the observed stimulation effects, we analyzed ECoG signals recorded from a strip electrode (AdTech) over the right sensorimotor cortex of a single patient undergoing the visuomotor task with their left arm (Fig. 4A). Time-resolved beta power between 20 and 35 Hz was extracted with Morlet wavelets from the channel over the motor cortex located closest to the central sulcus. To test for an effect of stimulation on oscillatory dynamics, while avoiding a potential bias from changes in movement speed, we pooled fast and slow trials with stimulation bursts and compared them to speed-matched trials without stimulation (Fig. 4B). Artifacts at the edges of the DBS burst resulting from switching DBS on and off were excluded. Comparison of stimulated to not-stimulated trial activity revealed a significant reduction in beta power in the perimovement window (0.1 to 0.3 s; *P* < 0.05). In contrast, analysis of the postmovement window showed a significant stimulation-induced increase in beta power after the cessation of stimulation (0.4 to 1 s; *P* < 0.05). These findings demonstrate that subthalamic DBS bursts as short as 300 ms can (i) instantaneously suppress beta oscillations in the motor cortex during movement and (ii) enhance postmovement beta rebound, previously suggested to relate to cortical reorganization and acute motor adaptation through learning (*27*–*29*). Notably, neither perimovement beta suppression nor postmovement beta enhancement was pro- or antikinetic per se, thus associated with changes in instantaneous kinematics (fig. S1). Instead, we interpret these findings as neurophysiological correlates of state-specific reinforcement of ongoing motor states.

### Toward fully embedded state-specific stimulation through movement speed decoding from invasive neurophysiology

Our behavioral finding showing a less pronounced bradykinetic decline from stimulation of fast compared to slow movements highlights the potential of acute motor state–specific DBS approaches for the treatment of PD in the future. The use of external sensors to infer behavioral states, however, such as the cursor position used in the present study, has limited practicality for chronic treatment of patients. Therefore, we aimed to investigate whether invasively recorded ECoG and local field potential (LFP) signals could be used to decode the motor behavior in the tablet task. The signals from five common average–referenced ECoG and two bipolar LFP channels were used to predict the movement speed of a single patient (maximum number of channels available; Fig. 4A). Six oscillatory features [alpha (8 to 12 Hz), low beta (13 to 20 Hz), high beta (20 to 35 Hz), low gamma (60 to 80 Hz), high gamma (90 to 200 Hz), and high-frequency activity (HFA) (200 to 400 Hz) power] and the raw amplitude were extracted from the signal using the real-time compatible pipeline provided by py_neuromodulation (*30*, *31*) (Fig. 4D). On the basis of these features, a gradient boosting machine learning model [CatBoost (*32*)] was trained to predict the continuously recorded movement speed of the patient (Fig. 4C). Only features from previous time points were used to inform the prediction of the instantaneous movement speed. The decoding performance of different channels and channel combinations was tested in a large-scale Bayesian optimization hyperparameter search (see table S3). A combination of all ECoG channels emerged with the highest performance (*R*^2^ = 0.38), similar to the combination of all available ECoG and LFP channels (*R*^2^ = 0.38). Notably, the best ECoG channel (*R*^2^ = 0.28) achieved higher decoding performances than both LFP channels combined (*R*^2^ = 0.15; Fig. 4C, average performance from an eightfold nested cross-validation). Analysis of feature importance revealed that alpha and low beta power estimates in the ECoG channel, located over the motor cortex, were the most informative for the decoding of movement speed (Fig. 4E). These results provide proof of principle that continuous movement speed can be decoded from motor cortex ECoG signals with high performance, which may pave the way for the development of fully embedded speed-dependent DBS approaches in the future.

## DISCUSSION

Our study implements a motor state-dependent closed-loop neurostimulation approach to provide an important proof of principle, showing that motor effects of STN-DBS depend on the acute motor state during which stimulation is applied. We derive three major findings from our study. First, we demonstrate that STN-DBS applied in brief bursts with closed-loop control does not have a prokinetic effect per se. Instead, we found that the same stimulation can evoke differential behavioral effects depending on concurrent behavior. Specifically, we found a more pronounced antibradykinetic effect by stimulating fast compared to slow movements, shifting the speed of the subsequent trial toward the speed of the stimulated movement, compatible with a reinforcement effect of closed-loop STN stimulation. Next, using normative functional connectomes, we elucidate brain-wide networks associated with this differential modulation, identifying the SMA and basal ganglia as key hubs for the induction of the state-dependent stimulation effect. Last, using intracranial cortex electrophysiology in a single patient, we report a stimulation-induced decrease in perimovement and an increase in postmovement cortical beta activity, suggesting that 300-ms STN-DBS modulates cortical processing associated with motor adaptation. In the same patient, sensorimotor ECoG signals could be used to decode continuous movement speed with high performance, supporting the feasibility of fully embedded movement-dependent DBS approaches. Together, we argue that the state during STN-DBS influences the behavioral aftereffects of stimulation, with stimulation of more vigorous motor behavior potentially providing a more effective alleviation of bradykinesia than stimulation applied during less vigorous motor states. This has important implications for the development of future DBS control algorithms, motivating a speed-adaptive approach for maximization of therapeutic efficacy.

### Reinforcement of behavior through basal ganglia neuromodulation: From rodents to humans

Traditionally, the basal ganglia have been divided into a prokinetic direct “Go” pathway and an antikinetic indirect “NoGo” pathway. Consequently, high-frequency STN-DBS, suppressing activity in the antikinetic indirect pathway, has been interpreted as being prokinetic per se. Recent works in rodents, however, challenge the dichotomy of solely pro- and antikinetic basal ganglia pathways by showing that both pathways are intrinsically active during movement initiation and that optogenetic stimulation of both pathways can invigorate or weaken movement (*10*, *33*). This bidirectional modulation was reported to depend on the concurrent movement state, with stimulation of direct striatal neurons leading to an increase in speed if fast and a decrease in speed if slow movements were stimulated (*10*). Studies modulating basal ganglia activity through manipulation of dopamine signaling further underline the state dependency of basal ganglia processing (*11*, *34*). Here, Markowitz *et al.* reported that exogenously induced dopamine transients in the dorsal striatum can reinforce the occurrence of the ongoing behavioral state in the future (*11*). Our study reports acute motor state–dependent modulation of motor behavior through STN-DBS in humans, indicating that its effect might go beyond a purely prokinetic mechanism and instead follow the logic of phasic dopaminergic control (*14*). Stimulation during fast movements resulted in a more positive modulation of movement speed than stimulation targeting slow movements. Looking more closely, we found that STN-DBS led to reinforcement-like shifts in future movement speed in a direction-specific and thus potentially neural pathway–specific manner. Although our findings are in line with the results from the speed-selective optogenetic stimulation in rodents, key differences merit further elaboration. As our study was performed with human patients with PD, we could not optogenetically stimulate striatal neurons but instead applied high-frequency STN-DBS. We argue that high-frequency STN-DBS may have a similar influence on net basal ganglia output as the activation of striatal pathway neurons (*14*). Here, optogenetic stimulation of D1-dopamine receptor–expressing neurons can shift the basal ganglia pathway balance away from the indirect pathway by increasing activity in the direct pathway. Similarly, STN-DBS can also modulate the pathway balance in favor of the direct pathway, not through activation of the direct pathway but through suppression of the subthalamic nucleus, the main driver of the indirect pathway (*12*, *35*, *36*). On the behavioral level, despite only stimulating a fraction of the experiment time, we observed lasting changes to movement speed in dependence on ongoing kinematics. How exactly this STN-DBS–induced modulation gives rise to changes in future motor behavior, however, can only be speculated and may involve plasticity at corticostriatal, thalamostriatal, thalamocortical, and corticocortical synapses (*14*, *37*, *38*). Another difference between our work and previous optogenetic works lies in the timing of stimulation. Although Yttri and Dudman triggered stimulation shortly after movement initiation based on the prediction of the peak speed, limited recording time and fluctuating baseline speed did not allow for sufficiently accurate prediction of peak speed in patients with PD, and thus stimulation was triggered after the peak speed. It has been reported that movement-related dopamine transients occur time-locked to movement onset (*39*, *40*); thus, modulation of basal ganglia activity during early movement periods could be even more effective in biasing future movements than the later window targeted in our study. Regardless of the underlying mechanism, our study provides evidence that motor effects of STN-DBS depend on ongoing movement kinematics and that bradykinesia, the cardinal feature of PD, may be best treated by stimulating during fast and vigorous motor output.

### Speed-selective STN-DBS engages brain-wide networks with the SMA and basal ganglia as key hubs

Over the past decade, MRI-based DBS connectomics has emerged as a powerful tool to uncover circuit effects of invasive neuromodulation in humans (*20*, *41*). Previous studies have focused on predicting patient-specific clinical or behavioral stimulation effects based on the individual location of chronically active stimulation electrodes (*17*, *20*, *42*). In our study, we extended this framework to the differential effect of state-specific stimulation algorithms, namely, the difference between stimulating fast versus slow movements. We could identify a whole-brain network related to the stimulation-induced speed-selective effect, elicited by only brief 300-ms bursts of closed-loop DBS. The more electrodes were connected to this network, the stronger their effect was on the identified movement-specific neuromodulation. The network highlighted broad frontal cortical, thalamic, and basal ganglia regions, which is in line with previously reported networks mediating clinical DBS effects (*17*, *43*). Beyond whole-brain connectivity, a region of interest analysis revealed that the SMA and basal ganglia nuclei best accounted for the observed effect. The relevance of these regions is in line with previous studies showing that the SMA (*44*), as well as the basal ganglia (*45*), encodes movement kinematics such as hand speed and that alterations in their activity are linked to bradykinesia (*46*). Moreover, both regions are involved in skill learning (*47*, *48*) and are known to drive changes in future motor behavior. In this regard, it has been hypothesized that “neural reinforcement” in cortico-basal ganglia circuits may refine and strengthen cortical activity patterns associated with successful behavior, ultimately driving skill learning and behavioral consolidation (*37*). Our findings can be integrated into this framework, as speed-selective STN-DBS may have reinforced neural patterns associated with either slow or fast movements, potentially associated with cortical beta oscillatory activity as discussed below.

### Speed-selective closed-loop STN-DBS modulates cortical beta power

To further elucidate the remote circuit effects of speed-selective STN-DBS, we investigated the impact of stimulation on motor cortex beta activity. In the subthalamic nucleus, excessive beta synchronization has been repeatedly linked to PD symptom severity, which is reduced by DBS (*4*, *49*, *50*). Although evidence focusing on cortical modulations is less consistent, work suggests that STN-DBS attenuates beta power over motor cortices (*51*). We extend this observation in a patient who received a contralateral ECoG strip by showing that <1-s STN-DBS applied during a movement reduces beta power recorded over the motor cortex. Moreover, we report that such short DBS bursts not only influence instantaneous oscillation but also increase postmovement beta synchronization, previously termed beta rebound (*52*). Although analysis of the present data from a single patient does not allow us to systematically investigate the relationship between cortical oscillatory changes and the strength of speed-dependent DBS effects, we may speculate about the involvement of postmovement beta rebound in the observed state-dependent effect. It has been reported that the strength of beta rebound is linked to the adaptation of motor behavior, with higher levels reflecting maintenance and reduced activity indicating an adaptation of the current motor program (*28*, *53*). Following this rationale, the increase in beta rebound after stimulation may be associated with the reinforcement and consolidation of ongoing activity patterns and thus increase the likelihood of producing a similar behavior in the future, i.e., moving fast after a fast movement has been stimulated and moving slow after a slow movement was stimulated. In our analysis, this effect was independent of the speed, as we have pooled trials from fast and slow movement speeds with stimulation and compared them to speed-matched trials without stimulation. Notably, although the stimulation-induced changes in cortical beta modulation were robust in the single-subject analysis, future studies in larger cohorts could reveal speed- and direction-specific neurophysiological underpinnings of motor state-specific stimulation paradigms, which were impossible to elucidate in the *n* = 1 experiment.

### Toward neural reinforcement with state-adaptive DBS for PD and beyond

Although chronic DBS has been an effective treatment option for PD for the past 30 years, a growing body of research focuses on the development of dynamically controlled adaptive DBS approaches, increasing treatment efficacy and reducing side effects. Besides more recent works suggesting adaptation based on gamma oscillations (*54*, *55*), most existing approaches for PD have focused on beta-band activity as a control signal, which has been associated with symptom severity (*6*). Although the clinical efficacy of beta-adaptive DBS has been reported (*56*) and its application is now being tested in clinical trials, concerns regarding the timing of stimulation have arisen. Subthalamic beta activity is reduced during movement (*57*, *58*), and consequently, beta-adaptive control reduces stimulation amplitudes during movement (*59*–*61*). Our data indicating reinforcement-like effects of STN-DBS motivates an alternative approach that focuses on the motor state as a potential control signal. Recently, Dixon *et al.* demonstrated the feasibility and efficacy of movement-adaptive DBS, increasing stimulation amplitude during a movement and decreasing the amplitude during rest periods (*62*, *63*). The idea of triggering DBS during selective behavioral states has also been explored for the treatment of tremor (*64*, *65*). These approaches do not rely on external sensors for the detection of motor states but, similarly to beta-adaptive approaches, decode the presence of these states from invasively recorded neural signals. Dixon *et al.*, for example, classified the occurrence of movement or rest on the basis of the oscillatory power in subthalamic LFP and cortical ECoG channels. Our results encourage the development of adaptive DBS algorithms beyond the binary classification of movement. Here, stimulation amplitude could be controlled on the basis of continuously decoded movement vigor to selectively stimulate and potentially reinforce high-vigor states in PD. It has been shown that grip force strength can be continuously decoded on the basis of subthalamic LFP and cortical ECoG signals (*66*). Similarly, we demonstrate in a single patient implanted with ECoG electrodes that the movement speed in the present task can be successfully predicted without the need for external sensors. The fact that we obtained the highest decoding performance using ECoG in addition to subthalamic local field potential (STN-LFP) signals aligns with a previous work showing the superiority of cortical over subcortical signals for movement decoding (*66*). Together, our findings motivate the development of speed-adaptive DBS approaches for the treatment of PD and provide a proof of principle that cortical ECoG may be a suitable decoding signal, allowing fully invasive closed-loop control. Beyond PD, reinforcement-like effects of DBS may be leveraged to accelerate learning outside the motor domain. For prosthetics such as brain-spine interfaces (*67*), auditory implants (*68*), and artificial retinas (*69*), state-selective DBS could, in the farther future, help to strengthen input-output relationships of neural circuits and thus accelerate adaptation to neuroprosthetics.

### Limitations

Although the abovementioned implications are promising, it is important to consider the limitations of this study. The primary aim of our study was to demonstrate that the effect of stimulation depends on the motor performance at the time stimulation is applied. We argued that the most rigorous way to do this was to directly compare two stimulation conditions that are defined by their difference in movement performance, which is why we have decided not to introduce additional alternative stimulation conditions, such as therapeutic chronic stimulation. Thus, a key limitation of this study is that we cannot infer the clinical efficacy of fast-selective DBS in comparison to traditional clinical chronic stimulation settings. In the future, a more clinical efficacy-oriented study design is required to clarify the clinical utility of our approach in comparison to chronic and other closed-loop DBS paradigms. Moreover, the strength of the observed speed-dependent stimulation effect varied between individuals, with only a subset of patients showing strongly elevated speed in the fast compared to the slow condition. Although a significant amount of this variation could be explained by differences in the stimulated volume, other factors might have contributed to the variability. Electrode implantation is known to induce a “stun effect,” characterized by a temporary suppression of parkinsonian symptoms, likely resulting from a microlesion in the subthalamic nucleus (*70*). Different extents of this “stun effect” across patients might have led to different states of the cortex–basal ganglia network and consequently to differential state-dependent DBS-induced processing. Another limitation of our study consists of the fact that only a preliminary clinical review could be carried out to select the stimulation parameters. Consequently, the strengths of the observed DBS effects may not fully represent the extent potentially obtainable after an extensive review of stimulation parameters. However, even with preliminary parameter optimization, the observed effects indicate a promising direction. Last, the results obtained from the ECoG signals in a single patient lack generalizability. Similarly, the discussed mechanisms are merely a speculation and must be interpreted cautiously. However, we argue that the presented results provide a valuable starting point for investigating state-dependent STN-DBS effects.

In conclusion, our study demonstrates that the motor state during which STN-DBS is applied critically influences its behavioral effects, providing a key proof of principle for reinforcement effects of state-dependent adaptive stimulation in PD. We show that closed-loop, motor state–dependent STN-DBS can differentially modulate behavior, with stimulation during faster movements exhibiting a more pronounced antibradykinetic effect. Stronger effect sizes were observed in electrodes with strong connectivity to the SMA and basal ganglia, whereas intracranial electrophysiology in a single patient revealed stimulation-induced changes in cortical beta activity. These findings underscore the potential and feasibility of reinforcement-based DBS strategies, suggesting that stimulation applied during more vigorous motor states may enhance therapeutic efficacy. Together, our results pave the way for the development of previously unknown DBS control algorithms that mimic dopaminergic reinforcement to optimize outcomes for individuals with PD and other dopaminergic disorders.

## MATERIALS AND METHODS

### Participants

Twenty-four patients diagnosed with idiopathic PD of primary akinetic-rigid motor phenotype with clinical indication for DBS were enrolled at the Department of Neurosurgery at the Charité–Universitätsmedizin Berlin (59.2 ± 9.4 years, five female; see table S1). We bilaterally implanted DBS leads into the subthalamic nucleus. In nine patients, we additionally implanted a subdural ECoG electrode strip unilaterally targeting the hand knob area of the primary motor cortex for research purposes. The experimental session was conducted in the perioperative state between the first and second surgical intervention and after the withdrawal of dopaminergic medication. Each patient underwent one session, including speed-selective stimulation during a behavioral task and simultaneous electrophysiological recordings. After completion of the experimental paradigm, we quantified the patient’s symptom severity with the Unified Parkinson’s Disease Rating Scale (UPDRS) III (29.0 ± 9.3; see table S1). In addition, we also recruited 14 healthy age-matched participants to perform the same behavioral task (59.5 ± 6.5 years, nine female; see table S1).

### Ethics declaration

The study presented in this manuscript was performed according to the standards set by the declaration of Helsinki and after approval by the ethics committee at Charité Universitätsmedizin Berlin (EA2/129/17). All patients and healthy participants provided informed consent to participate in the research. The data were collected, stored, and processed in compliance with the General Data Protection Regulation of the European Union.

### DBS and ECoG placement

DBS implantation followed a two-step approach. During the first surgery, we stereotactically placed DBS leads after coregistration of preoperative Magnetic Resonance Imaging (MRI) and Computed Tomography (CT) images. In nine patients, we additionally inserted a single ECoG electrode strip subdurally onto one hemisphere after minimal enlargement (~2 mm) of the frontal burr hole, directed posteriorly toward the hand knob region of the motor cortex and placed ipsilaterally to the implantable pulse generator (right = 8; left = 1). All electrodes were externalized through the burr holes via dedicated externalization cables. Patients remained on the ward for a duration of 4 to 7 days until the second surgery. During the second intervention, we replaced the externalization cables of the DBS leads with permanent cables, tunneled them subcutaneously, and connected them to a subclavicular implantable pulse generator. The ECoG electrodes were also removed at this stage.

### Anatomical localization of electrodes

We localized DBS and ECoG electrodes using standard settings in Lead-DBS (*71*). In brief, we linearly coregistered preoperative MRI and postoperative CT images using Statistical Parametric Mapping software (*72*) (SPM12; https://fil.ion.ucl.ac.uk/spm/software/spm12/), corrected them for brain shift, and normalized them to the MNI space (Montreal Neurological Institute; MNI 2009b NLIN ASYM atlas) using default presets of the Advanced Normalization Tools (*73*) (ANTs; http://stnava.github.io/ANTs/). DBS electrodes were automatically prereconstructed using the PaCER algorithm (*74*) for postoperative CT and later manually refined if needed. Last, we extracted MNI coordinates of each electrode contact for use in later analyses.

### Experimental paradigm

Rectangles of 175 x 175 pixels were displayed on a Wacom Cintiq 16 Tablet (1920 x 1080 pixels) using Psychtoolbox in MATLAB. Rectangles were placed either at the left end (100 pixels) or the right end of the tablet (1820 pixels) at one of the four positions along the *y*-axis (390, 465, 615, and 690 pixels). We instructed the patients to move a pen held in their dominant hand to the displayed rectangle. Upon arrival, the rectangle changed color and disappeared after an intermovement interval of 350 ms. At the same time, a rectangle on the opposite side appeared, leading to alternating movements from one side of the tablet to the other. Before the start of the paradigm, a short familiarization of 18 ± 6 trials was performed. This period served to instruct the patients to keep the pen on the tablet at all times, avoiding sampling errors from location jumps. The paradigm then consisted of four blocks containing 96 movements each. As rectangles were placed at four different locations along the *y* axis, 32 possible trajectories could be performed, which were pseudorandomized within each block and kept constant across participants. The cursor position was sampled at 62 Hz using MATLAB and used to calculate movement speed in real time. The difference between consecutive cursor positions was calculated, divided by the elapsed time, and averaged over six samples to obtain a smoother readout. Speed-selective stimulation was applied on the basis of the peak speed of each movement. The peak speed of the trial was extracted in one of the two ways: In 20 patients, the closest time point following the peak speed was detected by three subsequently decreasing speed values, and the maximum value of the previous samples was extracted as peak speed. In four patients, the time of peak speed after movement initiation was computed as the 80th percentile of 32 familiarization trials, and when reached, the peak speed was extracted for the ongoing movement. We used the former approach for most of the patients, as it reduced the number of additional trials while maintaining classification accuracy (see table S2 for group-averaged classification accuracy). To classify movements as slow or fast, the peak speed of the ongoing movement was compared to the peak speed of the previous two movements. A movement was classified as fast if the peak speed exceeded that of the previous two movements, and it was classified as slow if it fell below the previous two values. In blocks 1 and 3 (stimulation blocks), speed-selective stimulation targeted either slow or fast movements. We balanced the order of conditions across patients. Movements lasted 812 ± 115 ms on average. Stimulation, on average, started 66 ms after the peak speed and lasted 300 ms. In blocks 2 and 4 (recovery blocks), no stimulation was applied. A 30-s break was introduced between block 2 and block 3 for all patients, and an additional 30-s break between blocks 1 and 2, as well as 3 and 4, was present for 4 of 24 patients. Again, we used the former approach for most of the patients to reduce the overall recording time.

### Subthalamic deep brain stimulation

We implanted two different DBS lead models with either 8 (*n* = 23) or 16 (*n* = 1) contacts and used three segmented contacts on one level for monopolar stimulation. During a unilateral test stimulation preceding the stimulation paradigm, we progressively increased the amplitude and visually evaluated side effects as well as improvements in rigidity and movement speed of the UPDRS III finger tapping test. We tested all contacts, apart from the top and bottom ones. The contacts on each hemisphere with maximum stimulation amplitude and clinical improvement in the absence of side effects were selected for the subsequent experiment. We additionally evaluated side effects during bilateral stimulation and reduced stimulation amplitude if needed. During the experiment, stimulation was applied bilaterally without ramping at 130 Hz with a pulse width of 60 μs and a mean amplitude of 2.4 ± 0.4 (right) and 2.4 ± 0.5 (left) mA (see table S1 for details).

### Analysis of behavioral data

To extract kinematic measures specific to individual movements, the onset and offset of each movement had to be determined. After centering the data on the time point of peak speed, we defined movement onset and offset as the last samples above a threshold of 800.77 pixel/s. This threshold was defined as 3 SDs above the speed during the last 300 ms of the intertrial rest period aggregated over trials and patients. We then calculated the average movement speed between movement onset and offset as the main outcome measure for each trial.

For the analysis of speed modulation in a whole block, we excluded the first five trials from analysis, as well as trials whose average speed exceeded 2413.35 pixels/s, which were classified as outliers and replaced with NaN values. The outlier threshold was defined as 3 SDs above the average speed aggregated over trials and patients. To compare the change in speed between conditions, we normalized movement speed values with respect to the average value of the first five movements by subtracting and subsequently dividing by the average value, yielding the change in speed from block start in %. We also normalized speed values in the recovery blocks to the start of the preceding stimulation block. For statistical comparison, we averaged the speed values over trials for each block. For healthy control subjects, we averaged the speed values across both stimulation (1 and 3) and both recovery (2 and 4) blocks, as no stimulation was applied. We statistically compared the stimulation conditions in a within-subject design using a two-sided paired-sample nonparametric permutation test with 100,000 permutations at an alpha level of 0.05. To statistically compare the average change in speed between healthy control subjects and both stimulation conditions, we used a two-sided independent-sample nonparametric permutation test with 100,000 permutations at an alpha level of 0.05.

To analyze the effect of speed-selective stimulation on subsequent trials, we identified the stimulated and two subsequent movements for each subject. We then normalized the speed in the subsequent trials with respect to the stimulated trial, yielding a change of speed in %. As stimulated movements were either fast or slow, subsequent movement speed was shifted in the opposite direction, with speed decreasing after fast movements and increasing after slow movements. To compare stimulation effects independent of this inherent shift, we identified fast and slow movements in the recovery block, during which no stimulation was applied. Exactly as for stimulated movements, we calculated the change in speed of the two following movements. We then subtracted the change in speed following not-stimulated movements from the change in speed following stimulated movements. To statistically compare the difference in the stimulation-induced speed shift between conditions, we used a two-sided paired-sample nonparametric permutation test with 100,000 permutations at an alpha level of 0.05. To examine whether the speed shift for each condition significantly differed from 0, we performed a sign-flipping permutation test with 100,000 permutations at an alpha level of 0.05.

### fMRI-based functional connectivity analysis

We used an openly available PD fMRI group connectome (*17*) (www.lead-dbs.org) derived from the Parkinson’s Progression Markers Initiative (PPMI) database to investigate the relationship between speed-dependent stimulation effects and fMRI resting-state connectivity on the whole-brain level. All scanning parameters are published on the website (www.ppmi-info.org). We computed stimulation volumes using the Simbio/Fieldtrip model in the Lead-DBS toolbox for 23 of 24 patients (patient 4 was excluded because the implanted DBS lead model was not supported). Using the bilateral stimulation volumes as seeds, we generated whole-brain functional connectivity maps for each patient. Then, we correlated the connectivity values and the stimulation effects across patients for each voxel. This resulted in an R-map representing the whole-brain functional connectivity profile associated with the strongest stimulation effect. The stimulation effect was quantified as the difference between the average change in speed in % during blocks 1 and 2 (stimulation of fast movements and subsequent recovery) and the average change in speed during blocks 3 and 4 (stimulation of slow movements and subsequent recovery). We validated the explanatory power of the R-map using a leave-one-patient-out approach: For each patient, we calculated a distinct R-map based on the maps of the remaining patients, which was then spatially correlated with the connectivity map of the left-out patient. We then correlated the resulting network score, describing the similarity of the patient’s connectivity profile to the optimal map, with the observed stimulation effects. To investigate how individual brain regions contribute to explaining the variance of the observed stimulation effects, we further conducted a region of interest analysis. Using the bilateral stimulation as seeds, we extracted the average functional connectivity to 12 regions of interest, including the primary motor cortex, supplementary motor cortex, ventral anterior thalamic nucleus, parafascicular thalamic nucleus, globus pallidus externalis, globus pallidus internalis, substantia nigra pars reticulata, caudate nucleus, putamen, subthalamic nucleus, crus II cerebellum, and dentate nucleus cerebellum (*23*–*26*) for each patient. For each region, we used the average connectivity values to train a linear regression model predicting the stimulation effect. We then correlated the estimated effect with the empirical speed-dependent stimulation effect. We corrected *P* values using the FDR correction method (*75*). We used Spearman’s correlation for the calculation of R-maps and Pearson’s correlation for the quantification of explanatory power.

### Electrophysiology recordings

We recorded bilateral STN-LFPs using the remaining contacts and ECoG during the stimulation paradigm. Subdural ECoG strips were located contralaterally (*n* = 2) or ipsilaterally (*n* = 7) to the patient’s dominant hand, which was chosen for the behavioral task. All data were amplified and digitized with a Saga64+ (Twente Medical Systems International; 4000-Hz sampling rate) device. ECoG and STN-LFP signals were hardware referenced to the lowermost contact of the left DBS electrode. Data were saved to disk for offline processing and converted to the Brain Imaging Data Structure for iEEG data, principally in brainvision format (*76*). We used the data from patient 7, who performed the behavioral task contralaterally to the implanted ECoG strip, for exemplar analysis. We included five ECoG channels located in proximity to the sensorimotor cortices as well as five contralateral LFP channels in the analysis (see Fig. 4). We used custom Python scripts using MNE-Python (*77*), MNE-BIDS (*78*), NumPy (*79*), Pandas (*80*), and SciPy (*81*) for data processing and analysis (see the Data and materials availability section).

### Electrophysiological and behavioral data synchronization

We synchronized electrophysiology signals recorded with a SAGA64+ device and behavioral data recorded with MATLAB using a custom pipeline in Python (see the Data and materials availability section). We visually identified and marked the time point of the first DBS artifact using the lowest available LFP channel in the right hemisphere. Then, we identified the time point of stimulation onset in the behavioral data and subtracted the mismatch between electrophysiological and behavioral data from the behavioral data. Last, we upsampled the behavioral data recorded at ~62 Hz to the frequency of the electrophysiological recording (4000 Hz) by assigning the behavioral sample with the closest time stamp to each electrophysiological sample.

### Oscillatory power analysis

We used the signal recorded from the first ECoG electrode of patient 7, located over the motor cortex, to investigate changes in endogenous oscillations during STN-DBS. First, we rereferenced the signal by subtracting the average of all five ECoG electrodes (we excluded the sixth contact due to poor signal quality). Next, we epoched the data into 2-s windows centered on the time point of peak speed of each movement. Then, we transformed the data into the time-frequency spectrum using Morlet wavelets with four cycles and normalized using *z*-scores with respect to the 400 to 100 ms preceding the peak speed. To ensure that differences in oscillatory power between stimulated and not-stimulated movements were not attributed to differences in kinematics, we identified a sample of not-stimulated movements that had the same size and did not differ in peak speed from the sample of stimulated movements. For stimulated trials, we replaced the DBS edge artifacts with NaN values using the time point of stimulation onset/offset for each movement and a standardized window of 65 ms. For visualization purposes, we replaced these NaN values with linearly interpolated values. Then, we calculated the average beta power (20 to 35 Hz) during stimulation and after stimulation for each movement (0.13 to 0.3 s and 0.41 to 1.0 s, windows based on the average onset/offset of the DBS artifact). We statistically compared the beta power during stimulated versus not-stimulated trials using a two-sided independent nonparametric permutation test with 100,000 permutations at an alpha level of 0.05. Furthermore, we examined significant differences irrespective of predefined time bins using two-sided cluster statistics with 1000 permutations and an alpha level of 0.05. As this cluster analysis could not deal with NaN values and they were present during at least 1 epoch throughout the whole stimulation period, we replaced those values with the median of the remaining epochs if less than 15% of epochs contained NaN values, thus were affected by the DBS artifact. Time points during which more than 15% of epochs were affected were excluded from the cluster analysis.

### Movement speed decoding

We decoded the movement speed of patient 7 during the behavioral task using five ECoG contacts located over the contralateral sensorimotor cortices and five LFP contacts placed in the contralateral subthalamic nucleus. We rereferenced the ECoG signals by subtracting the average of all ECoG electrodes. For the LFPs, we summed the signals recorded from the second level of the DBS lead together and bipolarly referenced them against the lowest and highest contact, resulting in two LFP channels (1-2/3/4 and 8-2/3/4, with 1 being the lowest contact). To construct the target variable, we concatenated raw movement speed values across all task blocks. We calculated decoding features using the py_neuromodulation toolbox in Python and included alpha (8 to 12 Hz), low beta (13 to 20 Hz), high beta (20 to 35 Hz), low gamma (60 to 80 Hz), high gamma (80 to 200), and high frequency activity (HFA) (200 to 400 Hz) power computed by the fast Fourier transform and raw signal amplitude (*30*). The frequency at which the features were calculated and the length of the segment used for calculation were optimized in a Bayesian optimization hyperparameter search to achieve maximum decoding performance (see table S3). Furthermore, we *z*-scored the feature values to the features in the preceding 1 s and clipped them as an artifact rejection mechanism when they exceeded a value of [−3, 3]. We then used the machine learning algorithm CatBoost (*32*), which builds on the theory of decision trees and gradient boosting, to predict the movement speed at each sample on the basis of the instantaneous and a variable number of previous features. We set the number of boosting rounds to 30 and identified optimal tree depth, learning rate, and the number of preceding samples using a Bayesian optimization hyperparameter search (see table S3).

We tested the decoding performance of 10 different channel combinations (1 to 5: individual ECoG channels, 5 to 7: individual LFP channels, 8: combined ECoG channels, 9: combined LFP channels, and 10: combined ECoG and LFP channels) using rigorous nested cross-validation. For each channel combination, the data were split into folds of 87.5% training and 12.5% test set using an outer eightfold cross-validation. A Bayesian optimization search was conducted for 33 rounds using the training set only. For each optimization round, the training data from the outer loop was split into 75% training and 25% test set using an inner fourfold cross-validation. The mean coefficient of determination *R*^2^ averaged over all test sets of the inner loop was used to inform the hyperparameter search. We visualized decoding features for an exemplar ECoG channel and movement. Last, we extracted the feature importance, which measures how much the prediction changes when a feature is removed, from the CatBoost model trained on an exemplar outer fold with optimal parameters (the best model trained on all ECoG channels). Here, we averaged the importance of preceding samples for each channel and feature.
