# Supplementary material for: Differential modulation of movement speed with state-dependent deep brain stimulation in Parkinson’s disease
Source: Sci Adv. 2025 Sep 10;11(37):eadx6849. doi: 10.1126/sciadv.adx6849 (PMC12422180; doi:10.1126/sciadv.adx6849)
Supplement: Supplementary file 1 — Fig. S1 Tables S1 to S3 [file sciadv.adx6849_sm.pdf]

Science Advances
AAAS

Supplementary Materials for

Differential modulation of movement speed with state-dependent deep brain stimulation in Parkinson's disease

Alessia Cavallo *et al.*

Corresponding author: Wolf-Julian Neumann, julian.neumann@charite.de

Sci. Adv. **11**, eadx6849 (2025)
DOI: 10.1126/sciadv.adx6849

This PDF file includes:

Fig. S1
Tables S1 to S3

Fig. S1.

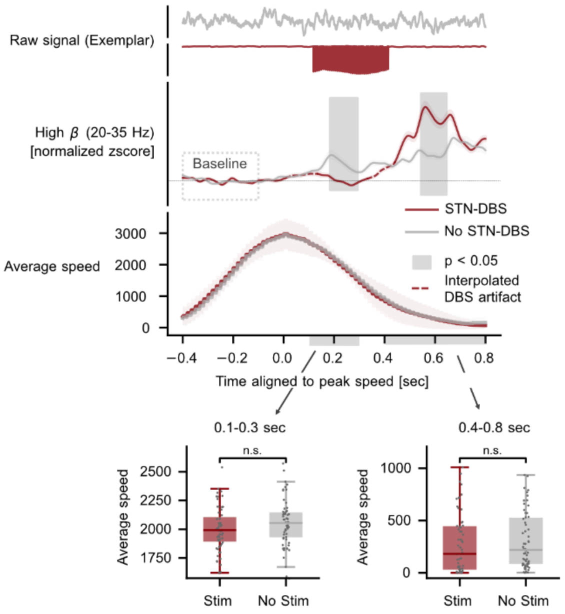

Comparison of average speed during stimulated and not-stimulated movement, related to figure 4. *While the comparison of motor cortex beta activity during stimulated and not-stimulated movements reveals a stimulation-induced decrease in beta power during the peri-movement and an increase during the post-movement period, comparison of average movement speed of the same movements shows no significant differences. Shaded light gray areas indicate clusters of significant differences. Shaded colored areas show the standard error of the mean. Differences between average movement speed of stimulated and not-stimulated trials during the peri-movement (0.1 – 0.3 s) and post-movement (0.4 – 0.8 s) period (gray bars below x-axis) are shown as box plots with trial-individual speed values. n.s. not significant*

Table S1.
Subject information

Healthy controls			**Parkinson's Disease patients**									
n	***Sex***	***Age***	**n**	**Sex**	**Age**	**Disease Duration**	**Days After Surgery**	**UPDRS_III OFF**	**Dominant Hand**	**Dominant Disease Side**	**DBS parameters**	**DBS lead model**
1	*f*	*55*	1	m	57	6	4	26	R	R	R2-, 2 mA, L3-, 2 mA, 60 ms, 130 Hz	MT SenSight Short
2	*m*	*61*	2	m	55	3	6	31	R	L	R2-, 1.5 mA, L2-, 1.5 mA, 60 ms, 130 Hz	MT SenSight Short
3	*f*	*56*	3	m	45	5	3	14	R	Equal	R3-, 3 mA, L3-, 3 mA, 60 ms, 130 Hz	MT SenSight Short
4	*f*	*68*	4	m	66	8	3	25	R	R	R3-, 2 mA, L3-, 2 mA, 60 ms, 130 Hz	BS Vercise Cartesia X
5	*f*	*68*	5	f	65	6	3	41	R	L	R2-, 2.5 mA, L2-, 2.5 mA, 60 ms, 130 Hz	MT SenSight Short
6	*f*	*62*	6	f	71	6	4	12	R	Equal	R3-, 2 mA, L3-, 2 mA, 60 ms, 130 Hz	MT SenSight Short
7	*m*	*69*	7	m	54	12	6	28	L	R	R3-, 2 mA, L2-, 2 mA, 60 ms, 130 Hz	MT SenSight Short

8	*f*	*48*	8	f	69	11	3	13	R	R	R3-, 2 mA, L3-, 2 mA, 60 ms, 130 Hz	MT SenSight Short
9	*m*	*51*	9	m	53	9	3	27	R	R	R2-, 2.5 mA, L3-, 2.5 mA, 60 ms, 130 Hz	MT SenSight Short
10	*f*	*64*	10	m	52	14	4	35	R	R	R2-, 2.5 mA, L2-, 2 mA, 60 ms, 130 Hz	MT SenSight Short
11	*f*	*64*	11	f	73	5	4	32	R	R	R2-, 2 mA, L2-, 2.5 mA, 60 ms, 130 Hz	MT SenSight Short
12	*m*	*53*	12	m	73	20	4	23	R	L	R2-, 2 mA, L3-, 1.5 mA, 60 ms, 130 Hz	MT SenSight Short
13	*f*	*54*	13	m	50	10	3	31	R	L	R3-, 2 mA, L3-, 2 mA, 60 ms, 130 Hz	MT SenSight Short
14	*m*	*60*	14	f	64	8	6	37	R	L	R2-, 2 mA, L2-, 3 mA, 60 ms, 130 Hz	MT SenSight Short
			15	f	62	6	2	33	L	R	R3-, 2.5 mA, L2-, 2.5 mA, 60 ms, 130 Hz	MT SenSight Short
			16	m	50	9	3	11	R	R	R2-, 2.5 mA, L2-, 2.5 mA, 60 ms, 130 Hz	MT SenSight Short
			17	m	68	6	6	40	R	R	R3-, 2.5 mA, L3-, 2.5 mA, 60 ms, 130 Hz	MT SenSight Short
			18	m	57	10	6	29	R	Equal	R2-, 3 mA, L2-, 2.5 mA, 60 ms, 130 Hz	MT SenSight Short
			19	m	41	20	6	44	R	L	R3-, 2.5 mA, L2-, 2.5 mA, 60 ms, 130 Hz	MT SenSight Short
			20	f	55	9	2	38	R	L	R2-, 3 mA, L2-, 2.5 mA, 60 ms, 130 Hz	MT SenSight Short
			21	m	43	10	6	38	R	L	R3-, 2.5 mA, L2-, 2.5 mA, 60 ms, 130 Hz	MT SenSight Short
			22	m	64	10	3	38	R	L	R3-, 2.5 mA, L2-, 3.5 mA, 60 ms, 130 Hz	MT SenSight Short
			23	f	72	4	4	29	R	R	R2-, 2 mA, L2-, 3.5 mA, 60 ms, 130 Hz	MT SenSight Short
			24	m	62	14	4	21	R	Equal	R2-, 3 mA, L3-, 3 mA, 60 ms, 130 Hz	MT SenSight Short
	9/15 F	*59.50*		8/24 F	60.50	8.79	4	26.79		9/24 L		
		6.511			8.95	4.28	1.13	8.57				

Table S2.

Speed-classification accuracy

	**Fast movement**	**Slow movement**
**Sensitivity**	91.9 ± 7.0 %	95.6 ± 9.1 %
**Specificity**	98.4 ± 1.9 %	96.2 ± 3.8 %

Table S3.

Bayesian Optimization Hyperparameters

**Feature extraction hyperparameters**	
Sampling frequency	20 to 50 Hz
Segment length	200 to 500 ms
**CatBoost Hyperparameters**	
Number of preceding samples	10 to 20
Learning rate	0.001 to 1
Tree depth	4 to 10
